# Osteosarcoma: Lessons Learned and Future Avenues

**DOI:** 10.1155/2013/641687

**Published:** 2013-09-04

**Authors:** Ajay Puri, Norman Jaffe, Hans Gelderblom

**Affiliations:** ^1^Department of Surgical Oncology, Tata Memorial Hospital, Mumbai 400012, India; ^2^Children's Cancer Hospital, University of Texas MD Anderson Cancer Center, Houston, TX 77030, USA; ^3^Department of Clinical Oncology, Leiden University Medical Center, Albinusdreef 2, 2300 RC Leiden, The Netherlands

Osteosarcoma is the most common malignant bone tumor in men. The incidence peaks around puberty, with a broader and lower peak after 60 years of age. Risk factors are (pubertal) growth, genetic factors, Paget's disease, and prior radiotherapy. More than 90% of tumors are of high grade, and their prognosis, even without metastasis at presentation, remains dismal in up to 30–45% of cases despite great improvements due to the introduction of chemotherapy some decades ago. 

The contribution “*Osteosarcoma: evolution of treatment paradigms*” to this osteosarcoma special issue is truly unique as it describes the history of systemic treatment from first hand, as the first author N. Jaffe coauthored the first studies on the dramatic improvement of long-term survival from 10–15% to 55–70% due to addition of multiagent chemotherapy. This magnitude of improvement has never been observed in other solid tumors, with the exception of germ cell tumors and some childhood cancers such as rhabdomyosarcoma and Ewing's sarcoma. However, these percentages have not further improved over the last decades, and new therapies are needed urgently to cure the remaining one-third of patients and increase the chances for patients with metastatic disease. The authors describe the design of the current EURAMOS study. Meanwhile, the first results of the PEG interferon randomization in the good responder arm were presented at the 2013 annual meeting of the American Society of Clinical Oncology [1]. From the 2260 included patients, 1034 had a good pathological response to preoperative chemotherapy. Of these patients 715, were randomized to either PEG interferon or observation. Only 76% of the patients randomized to interferon started treatment, mainly due to refusal. The event-free survival (primary endpoint) was not statistically improved in the interferon arm, although the results may have been influenced by the low randomization and high treatment refusal rate. Definite results and results of the poor responder randomization are pending. The study has proven that a worldwide platform for potential practice changing rapidly accruing randomized phase III studies is feasible in this rare disease and therefore should be used in the future.

The Birmingham experience with low-grade conventional osteosarcoma (LGCO), a rare (1.2%) and difficult to diagnose variant of osteosarcoma, is also part of this special issue. The diagnosis of LGCO is challenging due to the relatively nonspecific radiological and histological findings. Since treatment of LGCO is so different compared to a benign lesion as well as to high-grade osteosarcoma, accurate diagnosis is essential. The authors therefore advise that any difficult or nondiagnostic biopsies of solitary bone lesions should be referred to a specialist (bone) tumor unit for a second opinion: a conclusion that we fully agree with and should be part of all guidelines.

N. Federman et al. describe a novel osteosarcoma-associated cell surface antigen, ALCAM. The authors created an anti-ALCAM-hybrid polymerized liposomal nanoparticle immunoconjugate (*α*-AL-HPLN) to specifically target osteosarcoma cells and deliver a cytotoxic agent such as doxorubicin. If feasible in clinical practice, these *α*-AL-HPLNs are a promising new strategy to specifically deliver cytotoxic agents in osteosarcoma. A similar approach recently took place in breast cancer where the antibody-drug conjugate trastuzumab-DM1 (T-DM1) was designed to combine the biological activity of trastuzumab with the targeted delivery of a highly potent chemotherapeutic agent to HER2-overexpressing breast cancer cells. The success of the approach in breast cancer underlines the promise of *α*-AL-HPLN in osteosarcoma.

Y. Suehara et al. focus on proteomics to provide protein expression profiles of osteosarcoma that can be used to develop novel diagnostic and therapeutic biomarkers as well as to understand its biology. The authors provide a brief description of the methodology as well as examples of the recent proteomic studies that have generated new information regarding osteosarcomas. This approach should lead the way to predictive and prognostic information as well as necessary new drug targets in order to bring the necessary further improvement of our therapeutic strategies in osteosarcoma.

M. M. Hagleitner et al. show us a single center experience with osteosarcoma patients under the age of 20. In this retrospective series, improvement in toxicity and outcome was observed over the past 30 years that was attributed to improved supportive care allowing the intended full-dose chemotherapy regimen to be given. It is very well possible that improved experience due to centralization may also have added to this effect.

S. Piperdi et al. present a preclinical evaluation of the role of *β*-catenin in osteosarcoma development thought to originate from the mesenchymal stem cell. Despite fostering osteogenic differentiation, *β*-catenin does not induce the malignant features and tumorigenicity conveyed by oncogenic H-RAS when introduced into partly transformed mesenchymal stem cells. Despite this observation, C. H. Lin et al. show, using *in vivo *and *in vitro *studies, that Dickkopf-3 protein (Dkk-3) transfected 143B cells inhibited tumorigenesis and metastasis in an orthotopic xenograft model of OS. As Dkk-3 is known to inhibit the canonical Wnt/*β*-catenin pathway and its expression has been shown to be downregulated in osteosarcoma cell lines, we must realize that a delicate interplay of this pathway is present in osteosarcoma and requires further understanding before it can be targeted in the clinic.

In the paper by R. Muff et al., forty-eight common genes that are differentially expressed in metastatic cell lines compared to parental cells were identified. This subset of metastasis relevant genes in osteoblastic osteosarcoma overlapped only minimally with differentially expressed genes in the other four preosteoblast or nonosteoblastic cell line systems. These studies add to the microarray studies that were performed in the clinical research setting [2].

X. Mu et al. present a preclinical rationale for m-TOR inhibition for the treatment and prevention of osteosarcoma metastases. A phase II study including osteosarcoma patients was promising [3]. However, the Sarcoma mUltiCenter Clinical Evaluation of the Efficacy of riDaforolimus (SUCCEED) trial did not lead to registration of maintenance mTOR-targeted therapy in metastatic (osteo)sarcoma as the FDA rejected the application in May 2012 [4]. Maybe earlier treatment in nonmetastatic patients or combination treatment or patient selection based on prospectively collected biomarkers may lead the way to future clinical use.

In our opinion, the holy grail towards further survival benefit in osteosarcoma is thorough preclinical studies leading to new targets and biomarkers, followed by properly designed studies that can be performed rapidly in international collaboration of bone centers. This osteosarcoma issue of Sarcoma is just a tiny step in this process that will need perseverance.

*Ajay Puri* *Ajay Puri* *Norman Jaffe* *Norman Jaffe* *Hans Gelderblom* *Hans Gelderblom*
